# Discrepant Results of Experimental Human Mesenchymal Stromal Cell Therapy after Myocardial Infarction: Are Animal Models Robust Enough?

**DOI:** 10.1371/journal.pone.0152938

**Published:** 2016-04-06

**Authors:** Melina C. den Haan, Vanessa-Leigh van Zuylen, Niek J. Pluijmert, Cindy I. Schutte, Willem E. Fibbe, Martin J. Schalij, Helene Roelofs, Douwe E. Atsma

**Affiliations:** 1 Department of Cardiology, Leiden University Medical Center, Leiden, the Netherlands; 2 Department of Immunohematology and Blood Transfusion, Leiden University Medical Center, Leiden, the Netherlands; Emory University School of Medicine, UNITED STATES

## Abstract

**Background:**

Human mesenchymal stromal cells (MSCs) have been reported to preserve cardiac function in myocardial infarction (MI) models. Previously, we found a beneficial effect of intramyocardial injection of unstimulated human MSCs (uMSCs) on cardiac function after permanent coronary artery ligation. In the present study we aimed to extend this research by investigating the effect of intramyocardial injection of human MSCs pre-stimulated with the pro-inflammatory cytokine interferon-gamma (iMSCs), since pro-inflammatory priming has shown additional salutary effects in multiple experimental disease models.

**Methods:**

MI was induced in NOD/*Scid* mice by permanent ligation of the left anterior descending coronary artery. Animals received intramyocardial injection of uMSCs, iMSCs or PBS. Sham-operated animals were used to determine baseline characteristics. Cardiac performance was assessed after 2 and 14 days using 7-Tesla magnetic resonance imaging and pressure-volume loop measurements. Histology and q-PCR were used to confirm MSC engraftment in the heart.

**Results:**

Both uMSC and iMSC therapy had no significant beneficial effect on cardiac function or remodelling in contrast to our previous studies.

**Conclusions:**

Animal models for cardiac MSC therapy appear less robust than initially envisioned.

## Introduction

Cardiovascular disease (CVD) is the main mortality cause in the western world [1]. Over the past decade, studies have explored the potential of cell therapy as a novel treatment option for coronary artery disease. Various cell types including bone marrow-derived mononuclear cells [2], human mesenchymal stromal cells (MSCs) [3–5], embryonic stem cells [6], induced pluripotent stem cells [7], skeletal myoblasts [8] and cardiac progenitor cells [9] have been studied, showing varying results in both animal models and in patients [10–12]. MSCs constitute an attractive therapeutic cell type in view of their immunomodulatory and anti-inflammatory properties, easy expandability and their ability to support tissue regeneration [13,14]. The reported improvement in heart function by MSC administration seems mostly attributed to paracrine mechanisms [15], leading to neoangiogenesis [16], anti-apoptotic effects [17] and attenuation of the ventricular remodelling process [18]. Previously, we reported beneficial therapeutic effects of injection of unstimulated human MSCs (uMSCs) from CVD patients in a myocardial infarction (MI) model in NOD/*Scid* mice [4,19]. In the present study we aimed to extend this research by assessing the effect of pre-stimulating the human MSCs (iMSCs) with the pro-inflammatory cytokine interferon gamma (IFN).

Recent studies in animal models of graft–versus-host disease (GVHD) and colitis indicated that pre-stimulation of MSCs with IFN enhances their therapeutic effect [20,21]. The mechanism of this reported therapeutic benefit is not known but the role of immunomodulatory proteins that are upregulated by IFN including indoleamine 2,3 dioxygenase (IDO) and inducible nitrix oxide synthase, is currently being investigated [20–25]. We hypothesized that IFN pre-stimulation of MSC also enhances their beneficial effect on cardiac function in a NOD/*Scid* model of MI. Also, as MI causes the influx of inflammatory cells into the injured myocardium [26], we investigated whether the inflammatory cell influx was altered by uMCs and iMSCs administration.

## Materials and Methods

See S1 File for extended materials and methods.

### Animals

All experiments were approved by the Committee on Animal Welfare of the Leiden University Medical Center (LUMC) and conformed to the *Guide for the Care and Use of Laboratory Animals* as stated by the U.S. National Institutes of Health. To avoid rejection of transplanted human cells, 8- to 10-weeks-old male NOD/*Scid* mice (Charles River Laboratories, Maastricht, the Netherlands) were used.

### Primary cultured MSCs

Bone marrow aspiration procedures were performed in accordance with the Helsinki Declaration and were approved by the ethics committee of LUMC. All MSC donors provided written informed consent. Bone marrow-derived MSCs were obtained from three non-cardiac patients undergoing orthopedic surgery. IFN stimulation of MSCs was performed by adding 500U/ml IFN (Sigma-Aldrich Chemie BV, Zwijndrecht, the Netherlands) to the culture medium for 7 days.

Immunophenotyping of cultured MSC was performed using the following primary antibodies: CD90, CD73, MHC-I, CD34, CD45, CD31, CD80, HLA-DR (BD Biosciences, San Diego, USA), and CD105 (Ancell Corp., Bayport, MN, USA). MSCs from passages 4 to 5 were used for transplantation experiments after lentiviral transduction with a human vector expressing the enhanced green fluorescent protein (eGFP) gene. The cells transduced with lentivirus for eGFP, transmitted the eGFP signal in the FITC channel of the FACSCanto II (BD Biosciences, San Diego, CA, USA).

#### *In vitro* differentiation

To test the ability of uMSCs and iMSCs to differentiate into osteogenic and adipogenic lineages, cells were incubated in appropriate differentiation media as described previously [20]. MSCs were stained for alkaline phosphatase activity with Fast Blue (Sigma-Aldrich Chemie BV, Zwijndrecht, the Netherlands) and for calcium deposition with Alizarine Red (MP Biomedicals LLC, IllkirchCedex, France.) Formation of lipid droplets was visualized with Oil-red O staining (Sigma-Aldrich Chemie BV, Zwijndrecht, the Netherlands).

#### Suppression of PBMC proliferation by MSCs

Cultured MSCs were plated in graded doses in 96-well flat-bottom plates (Corning, Life Sciences). Human peripheral blood mononuclear cells (PBMC) isolated from buffy coats (1.0 x 10^5^/well) were added to the MSCs and stimulated with human T-activator CD3/CD28 dynabeads (Invitrogen Corp., Paisley, UK) in a bead:cell ratio 1:5. The cultures were harvested on a glass fiber filter and thymidine incorporation was measured with a liquid scintillation counter (Wallac, Turku, Finland).

### MI induction and Cell injection

Mice received buprenorphine subcutaneously before surgery and again 12 hours after surgery. MI was induced as described previously [9]. Briefly, animals were anesthetized with 5% isoflurane for induction, subsequently intubated and kept anesthetized with 1.5–2% isoflurane in oxygen for the remainder of the surgical procedure. After a left thoracotomy, the left anterior descending (LAD) coronary artery was ligated 1 mm caudally from the tip of the left auricle using a 7-0-prolene suture (Johnson and Johnson, New Brunswick, NJ, USA). Five minutes after LAD ligation the animals received either 2×10^5^ uMSCs in 15 μL phosphate buffered saline (PBS) (uMSC group), 2×10^5^ iMSCs in 15 μL PBS (iMSC group) or 15 μL PBS containing no cells (PBS group). Intramyocardial injections were performed at 3 sites in the infarcted area (5 μL per site). Sham-operated animals were operated in parallel to determine baseline characteristics (Sham group).

### Cardiac Magnetic Resonance Imaging (MRI)

Cardiac parameters were assessed 2 and 14 days post-MI using a 7-Tesla MRI (BrukerBiospin, Ettlingen, Germany). Mice were pre-anesthetized as described above and kept anesthetized with 1.5–2% isoflurane in oxygen for the remainder of the procedure. All data were analysed with the MASS for Mice software package (Leiden, the Netherlands). The endocardial and epicardial borders were delineated manually (uMSC group n = 12, iMSC group n = 7, PBS group n = 7, Sham group n = 10), after which left ventricular (LV) end-diastolic volume (EDV), LV end-systolic volume (ESV) and ejection fraction (EF) were computed.

### Pressure-Volume (PV) measurements

Fifteen days after MI, mice were anesthetized again as described above and kept anesthetized with 1–1.5% isoflurane in oxygen for the remainder of the procedure. A 1.2F pressure-conductance catheter (ScisenseInc, London, Canada) was introduced via the right carotid artery and positioned in the LV. The conductance catheter was connected to a PV control unit FV 896B (ScisenseInc, London, Canada). Parallel conductance and LV pressure-volume signals were measured as described previously [9]. All data were acquired using Powerlab 8/30 Model ML870 (ADInstruments, Spechbach, Germany) and LabChart 7 software (ADInstruments, Spechbach, Germany). Data were analyzed off-line (uMSC group n = 7, iMSC group n = 7, PBS group n = 5, Sham group n = 7).

### Histology

At day 15 post-MI, mice were weighed, sacrificed after PV loop measurements and hearts and lungs were removed. Lungs were weighed immediately after excision, freeze-dried for 24 hours and then weighed again. The wet weight/dry weight ratio was used as a measure of pulmonary congestion.

Per group 5 hearts were fixed by immersion in buffered 4% paraformaldehyde and embedded in paraffin. Serial transverse sections of 5 μm were cut along the entire long axis of the LV for (immuno)histological analyses. MSC engraftment was detected by immunostaining with a rabbit anti-GFP antibody (A11122, Invitrogen, Paisley, UK), followed by a biotinylated goat anti-rabbit IgG(E0432, Dako, Glostrup, Denmark) and a Qdot 655 streptavidin-conjugated (Q10121MP, Invitrogen, Paisley, UK) antibody.

### Real-time PCR

Per group, 5 hearts were used for DNA extraction to determine MSC engrafment rate by quantification of human genomic DNA in mouse hearts [27]. DNA concentrations were measured using NanoDrop 1000 (NanoDrop products, Wilmington, DE, USA). PCR reactions were performed in a volume of 10 μL, containing 5 μL Universal PCR Master Mix (Applied Biosystems, Carlsbad, CA, USA), 900 nM forward and reverse primers, 250 nMTaqManprobe and 50 ng of target template. Standard curves were generated by serially diluting human genomic DNA (Roche, Basel, Switzerland) in murine genomic DNA.

### Flow cytometry

From each treatment group, mice not subjected to functional and histological analysis were used for flow cytometric analysis of cardiac inflammatory cell invasion. Mice were sacrificed on days 1, 3, and 7 after MI. Each time point, 3 hearts were harvested per group. Sham animals were used as controls to determine base line characteristics. Total cardiac cell numbers were determined with a Sysmex cell counter (Sysmex America, Inc. Mundelein, Illinois, US). Single-cell suspensions were stained for flow cytometry with primary antibodies before analysis using a FACSCanto II (BD Biosciences, San Diego, CA, USA). The following antibodies were used: anti–CD90-APC, 53–2.1,–B220-APC, RA3-6B2,–CD49b-APC, DX5,–NK1.1-APC, PK136,–Ly-6G-APC, 1A8, CD11b-eFluor 450, M1/70,–CD11c-FITC, HL3,–I-A^b^ -FITC, AF6-120.1,–Ly-6C-PE, AL-21,–CD11c-PE, HL3 (All above antibodies are from BD Biosciences),–F4/80-FITC, C1:A3-1 (ABD Serotec, Kidlington, UK). Monocytes were identified as CD11b high (CD90/B220/CD49b/NK1.1/Ly-6G) low (F4/80/I-A^b^ /CD11c) low Ly-6C high/low as previously described [26,28]. Macrophages were identified as CD11b high, F4/80 high. Dendritic cells were identified as CD11b, I-A^b^andCD11c high. Neutrophils were identified as CD11b, Ly-6G high. The analysis of the acquired data was done with FlowJo software version 7.6.1 (Tree Star Inc. Ashland, OR, USA).

### Statistical Analysis

Numerical values were expressed as means ± standard deviation (SD). Comparison of MRI parameters and inflammatory cells between the uMSC, iMSC, PBS and Sham groups was performed using two-way repeated-measures analysis of variance (ANOVA), with Bonferroni correction. Comparison of the remaining parameters was performed using one-way ANOVA with Bonferroni correction.

## Results

### Characterization of MSCs

**Fig 1:**
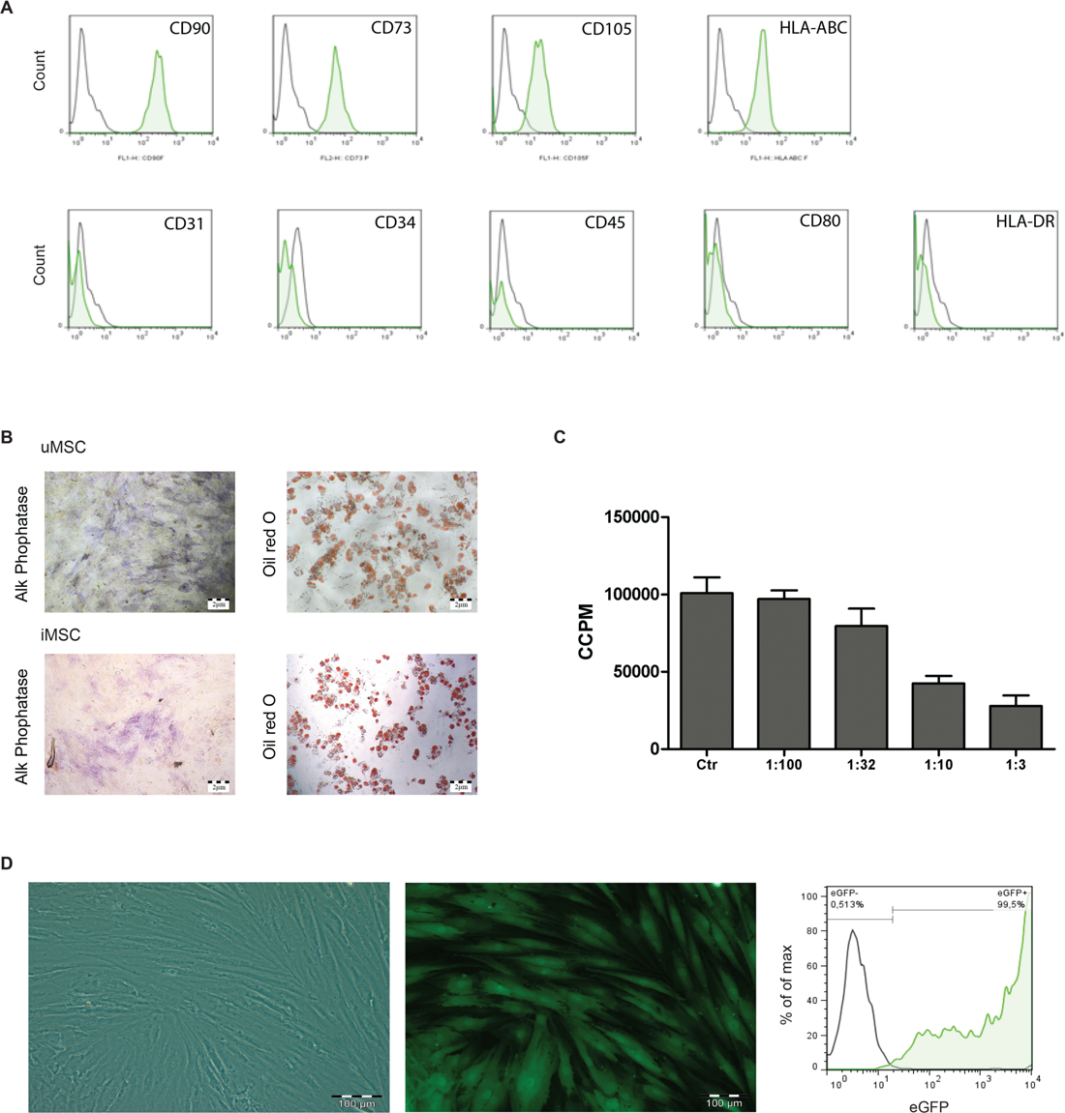
Characterization of primary cultured MSCs. A: *In vitro* characterization of MSCs consisting of the specific surface marker antigen panel measured by flow cytometry. B: The differentiation capacity of MSCs towards osteoblasts shown by alkaline phosphatase activity and towards adipocytes shown by lipid droplets staining via Oil Red O. C: The inhibitory capacity of proliferation of activated peripheral blood mononuclear cells measured by 3H-thymidine uptake in counts per minute (CCPM). D: Bright light and fluorescence microscopy and flow cytometric analysis of eGFP labeling of lentivirally transduced MSCs.

The uMSCs *in vitro* expressed the established surface marker profile: CD90+, CD73+, MHC-I+, CD105+, and CD45-, CD34-, CD80- (Fig 1A). In addition, all (u/i)MSCs were able to differentiate into osteoblasts and adipocytes (Fig 1B) and demonstrated an inhibitory capacity on PBMC proliferation (Fig 1C), confirming the definition and behavior of true MSCs).

The transduction efficiency of the (u/i)MSCs used was 100% which was analyzed by bright light microscopy and flow cytometry *(*Fig 1D).

**Fig 2:**
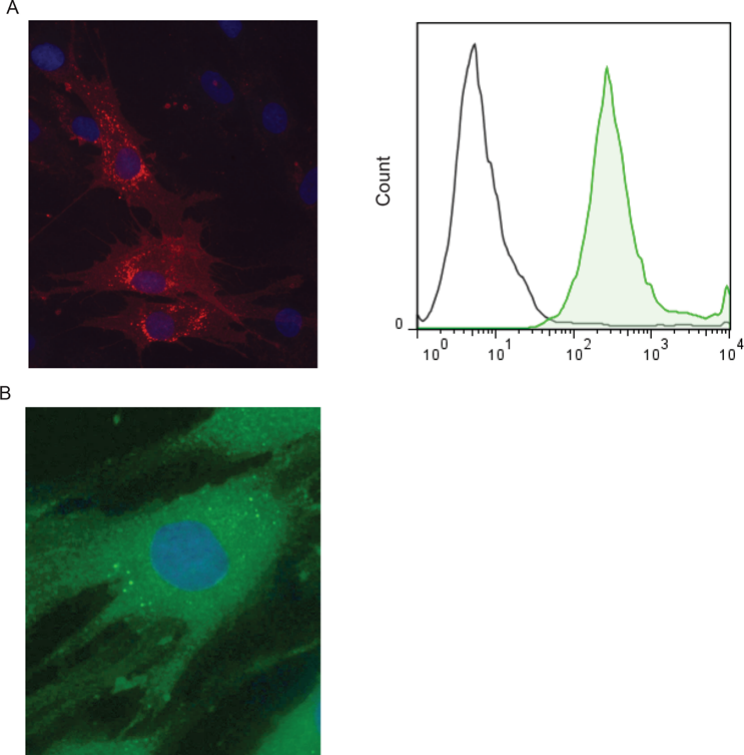
Stimulation of MSCs with the pro-inflammatory cytokine interferon gamma (IFN). A: From left to right: Immunostaining of iMSCs for HLA-DR in red (PE) and nuclei in blue (DAPI) and expression of HLA-DR as measured by flow cytometry. B: Immunostaining of iMSCs for the immunomodulatory enzyme IDO in green (ALEXA 488 nm) and nuclei in blue (DAPI).

After IFN stimulation, iMSCs expressed HLA-DR (Fig 2A), while the other expressed surface markers were comparable to uMSC (data not shown). The *in vitro* differentiation capacity toward osteoblasts and adipocytes was similar between iMSCs and uMSCs for all MSC sources used (data not shown). The IFN stimulation upregulated the expression of the intracellular enzyme indoleamine 2,3dioxygenase (IDO) as shown by immunohistochemistry (Fig 2B).

### Effect of MSC therapy on cardiac function

**Fig 3:**
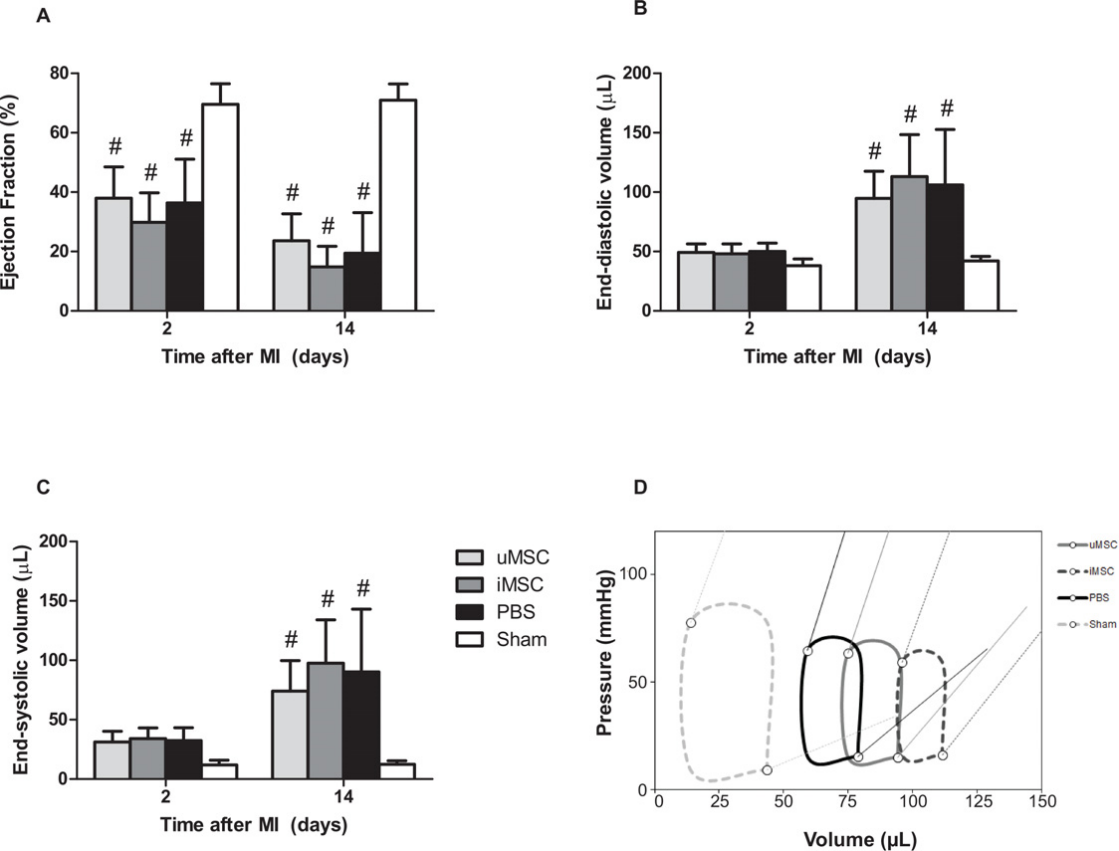
Cardiac function as assessed by 7 T MRI at 2 and 14 days after myocardial infarction in uMSC and iMSC treated animals (uMSC and iMSC, resp.), PBS treated animals (PBS) and Sham-operated animals (Sham) (panel A-C). A: Left ventricular ejection fraction. B: End-diastolic volume. C: End-systolic volume. Data are expressed as mean ± SD. # = p<0.05 versus Sham. D: Pressure Volume loops of all treatment groups at day 15 after MI. The oblique lines represent the end-systolic (Ees) and end-diastolic (Eed) pressure–volume relations.

Two and 14 days after inducing MI, LVEF decreased in all MI groups when compared to Sham as assessed by MRI (Fig 3A). The EDV and ESV were significantly increased 14 days after MI in all MI animals (Fig 3B and 3C). There were no differences between the uMSC group, the iMSC group or the PBS group for either EF, EDV or ESV at 2 or 14 days post MI.

These findings were confirmed by PV loop measurements 15 days after MI. LV EDV and ESV were significantly increased in all animals subjected to MI when compared to Sham, revealing substantial cardiac dilatation after MI. End-systolic pressure was significantly decreased in all MI groups indicating a reduced LV function, while the end-diastolic pressure was comparable for all four groups *(*Fig 3D*)*. There were no differences in parameters between the uMSC, iMSC and the PBS group, indicating no beneficial effect of the uMSC or iMSC treatment on cardiac function.

### Engraftment

**Fig 4:**
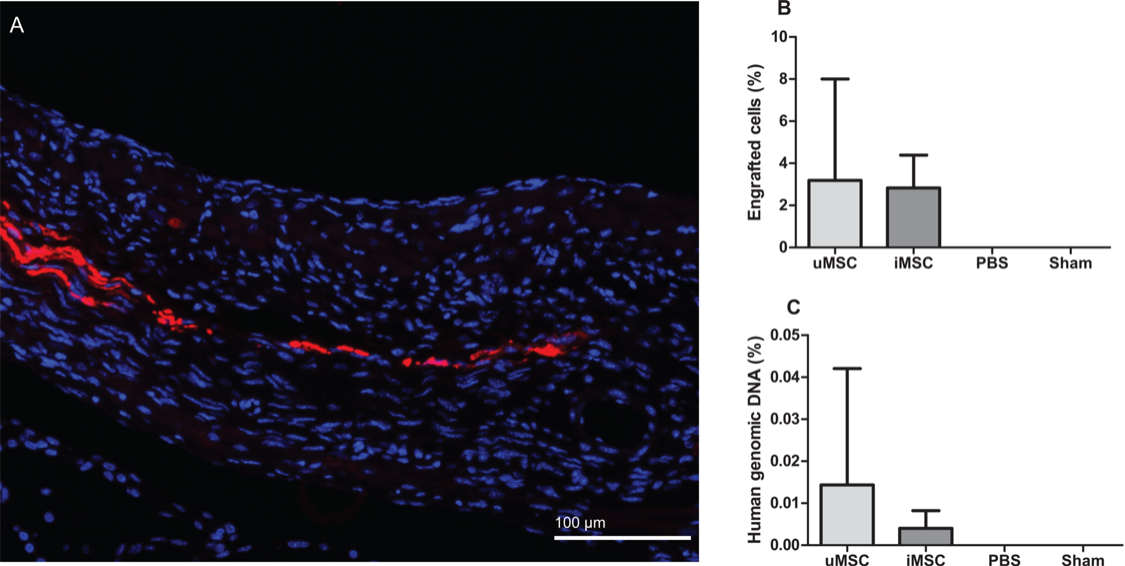
Assessment of engraftment of injected MSCs in mouse hearts 15 days after injection into the infarcted myocardium. A: Immunofluorescent staining of engrafted eGFP-labeled MSCs in red (Qdot 655) and nuclei in blue (hoechst 33342). B: Histological quantification of uMSC and iMSC engraftment (uMSC and iMSC resp.). PBS treated animals (PBS) and Sham-operated animals (Sham) were used as controls. C: Quantitative PCR for human genomic DNA in mouse hearts in animals treated with uMSC, iMSC, PBS and sham-operated animals. Data are expressed as mean ± SD. There were no significant differences between the uMSC and iMSC group.

Histological analysis showed that both uMSCs and iMSCs were engrafted into the myocardium at 15 days after injection, predominantly in the infarcted anterolateral wall *(*Fig 4A*)*. Quantitative assessment of histological sections revealed similar engraftment rates for the two cell groups (3.2% and 2.8% of injected cells for uMSC and iMSC, respectively, Fig 4B*)*. In addition, we performed q-PCR for human ALU repeats to determine cell engraftment of the human MSCs in the mouse hearts. q-PCR showed a small, but not significantly different, percentage of human genomic DNA in both uMSC and iMSC treated animals (Fig 4C*)*. Tissue from mice hearts injected with PBS and Sham-operated animals were used as control.

### Pulmonary congestion and body weight

**Fig 5:**
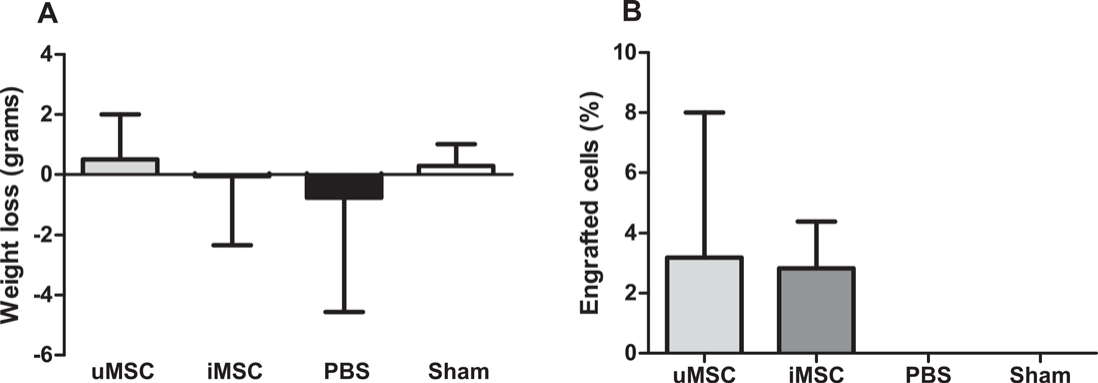
Physical parameters 15 days after myocardial infarction in uMSC and iMSC treated animals (uMSC and iMSC, resp.), PBS treated animals (PBS) and Sham-operated animals (Sham). A: Weight loss. B: Amount of pulmonary fluid. Data are expressed as mean ± SD. There were no significant differences between the uMSC, iMSC, PBS and Sham group.

Body weight was similar in all animals prior to surgery and did not significantly change between MI groups and the Sham-operated animals at day 15 post MI *(*Fig 5A*)*. In addition, no significant differences were observed in the amount of lung fluid between all MI groups and the Sham group *(*Fig 5B*)*.

### Inflammatory cell influx in the infarcted heart

**Fig 6:**
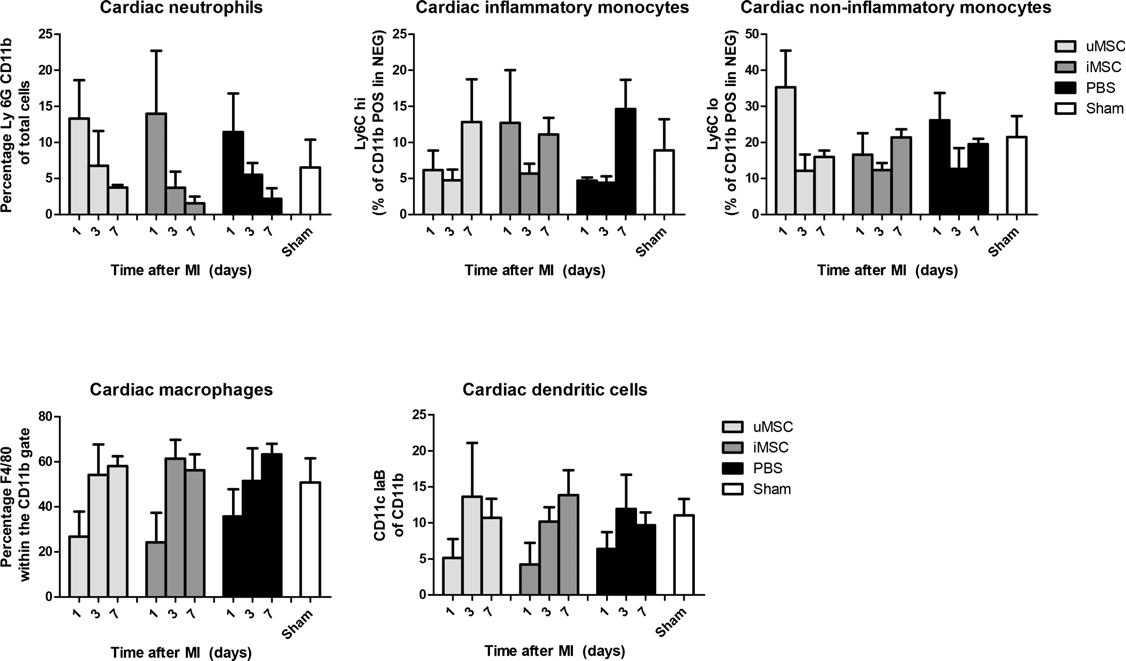
Flow cytometric analysis of inflammatory cells in the heart 15 days after myocardial infarction in uMSC and iMSC treated animals (uMSC and iMSC, resp.), PBS treated animals (PBS) and Sham-operated animals (Sham). Data are expressed as mean ± SD. There were no significant differences between the uMSC, iMSC, PBS and Sham group.

In the MI groups the frequency of inflammatory cells in the heart did not significantly deviate from the sham group. However, a trend of increased influx of neutrophils and non-inflammatory monocytes was found and a decreased presence of inflammatory monocytes, macrophages and dendritic cells on the first day after MI. This was not significantly different when comparing all MI groups to Sham animals. In addition, there appeared to be a trend of a decreased presence of inflammatory monocytes, macrophages and dendritic cells at day 1 after MI. However, this was also not significantly different when compared to Sham. Most importantly, no difference between the PBS group and either of the 2 MSC treatment groups or between the groups themselves could be shown (Fig 6*)*.

## Discussion

The main findings of this study are: (1) stimulation of human bone marrow-derived MSCs with IFN does not alter their differentiation capacity; (2) uMSCs and iMSCs engraft in infarct myocardium and (3) neither treatment with uMSC nor iMSCs improves cardiac function after MI. Although we hereby confirmed the engraftment rate of our earlier findings, we now had discordant results with regard to cardiac function [4,19].

In an attempt to explain why we could not reproduce our previous results, we compared the two study designs. For both studies, 8–10 week-old male NOD/SCID mice from Charles River Labs (Maastricht, NL) were used. Although physically at a different location, the mice were housed under identical conditions. The source of the MSCs used in the present study (orthopaedic surgery patients without known cardiac disease) differed from the MSCs we used previously (ischemic heart disease patients). However, the phenotypic (*in vitro* surface marker expression profile) and functional (osteogenic and adipogenic differentiation and immunomodulatory capacity) characteristics of the MSCs used in both studies were comparable. Non-cardiac patient derived-MSCs have also been tested in other experimental disease mouse models in which they showed therapeutic efficacy [20]. The presence of injected MSC in the cardiac tissue was confirmed in both studies 2 weeks after MI by histology and human *Alu* repeats-pcr. Also, the levels of MSC engraftment were comparable (3.2% in the current study vs 4.1% in our previous study [4]).

The operating procedures were performed by different, although thoroughly trained and experienced operators. Baseline EF, EDV and ESV 2 days after MI were comparable in the two studies, indicating similar myocardial damage and loss of cardiac function. In the current study we used a 7-Tesla MRI, while in our previous study a 9.4-Tesla MRI was used and measurements were performed by a different, although experienced operator. However, studies about inter-operator differences report of excellent reproducibility of the cardiovascular magnetic resonance measurements of the left ventricular volumes and mass [29,30].

All together, we have encountered unexplained discrepant therapeutic efficacy of MSCs in the permanent ligation MI mouse model. The experimental conditions were overall the same, suggesting that this discrepancy is related to details that are not recognized as potential key parameters. Therefore, questions arise concerning the robustness of these intricate animal models to assess the efficacy of cell therapy using human cells.

Several other studies, both positive and negative, have been performed investigating the therapeutic potential of MSC infusion in cardiac disease models. These studies showed therapeutic benefit on cardiac function mostly via echocardiography or MRI and whilst the experimental protocols seem similar between studies, large variations exist between the reported parameters that were beneficially influenced by MSC infusion, which hampers a direct comparison between studies [5,18,31–36].

In contrast, debate exists about the therapeutic efficacy of MSC therapy in various experimental cardiac disease models, fuelled by a growing number of negative studies on MSC therapy [12,17,37–41]. In a direct comparison between intramyocardial injection of various adult cell types in a mouse model of MI, using bone marrow-derived MSCs, bone marrow-derived mononuclear cells, skeletal myoblasts and fibroblasts, the MSCs did not show any beneficial effect on the preservation of cardiac function while the bone marrow-derived mononuclear cells did [12]. Another study by *van der Bogt et al* confirmed this result in a comparable study of adipose tissue-derived MSC and bone marrow-derived MSCs where neither cell type was able to preserve cardiac function [37].

Our current results indicate that the reported therapeutic effects of MSCs may be easily obscured by unknown study parameters. This poses a major limitation on the use of animal models in further development of cell therapy for cardiac diseases.

## Conclusion

Both uMSC and iMSC therapy have no significant beneficial effect on cardiac function or remodelling in a NOD/scid mouse model of myocardial infarction, in contrast to our previous studies. Animal models for cardiac MSC therapy appear less robust than initially envisioned.

## Supporting Information

S1 File (DOCX)
