# Supplementary material for: Discrepant Results of Experimental Human Mesenchymal Stromal Cell Therapy after Myocardial Infarction: Are Animal Models Robust Enough?
Source: PLoS One. 2016 Apr 6;11(4):e0152938. doi: 10.1371/journal.pone.0152938 (PMC4822837; doi:10.1371/journal.pone.0152938)
Supplement: S1 File — (DOCX) [file pone.0152938.s001.docx]

**Extended Materials and Methods**

**Animals**

All experiments were approved by the Committee on Animal Welfare of the Leiden University Medical Center and conformed to the *Guide for the Care and Use of Laboratory Animals* as stated by the U.S. National Institutes of Health. To avoid rejection of transplanted human cells, 8- to 10-weeks-old male non-obese diabetic/severe combined immunodeficient (NOD/*scid*) mice (Charles River Laboratories, Maastricht, the Netherlands) were used.
All animals were housed in filtertop cages and were given standard diet and water with antibiotics and antimycotics *ad libitum.*

**Primary cultured human Mesenchymal Stromal Cells**

Bone marrow derived MSC (BM-MSC) were obtained from patients undergoing orthopedic surgery. The bone marrow mononuclear cells (MNC) were isolated by Ficoll-Paque density gradient centrifugation (density: 1.077 g/cm3) and were plated at 1.3 x 10^5^/cm^2^ in DMEM-low glucose (DMEM-LG; Invitrogen Corp., Paisley, UK) supplemented with 10% fetal calf serum (FCS; Greiner Bio-one) and Penicillin/Streptomycin (P/S; Invitrogen Corp., Paisley, UK). Cultures were grown in 175 cm^2^ flasks (Corning Life Sciences B.V., Schiphol-Rijk, The Netherlands) in a 37°C humidified incubator containing 5% CO_2,_ and the medium was refreshed every 3-4 days. When the spindle shaped MSC monolayer reached >80% confluence, cells were detached using trypsin/EDTA (Invitrogen Corp., Paisley, UK) and replated at a density of 4,000 cells per cm^2^. IFN-γ stimulation of MSC (iMSC) was performed by adding 500U/ml IFN-γ (Sigma-Aldrich Chemie BV, Zwijndrecht, The Netherlands) to the culture medium for 7 days, refreshing the medium within 3 days.

Immunophenotyping of cultured MSC (both uMSC and iMSC) was performed using the following primary antibodies: CD90, CD73, MHC-I, CD34, CD45, CD31, CD80, HLA-DR (BD Biosciences, San Diego, USA), and CD105 (Ancell Corp., Bayport, MN, USA). Samples were analyzed using a FACSCalibur flow cytometer (BD Biosciences, San Diego, CA, USA) and the data were analyzed with FlowJo software (version 7.6.3., Tree Star Inc. Ashland, OR, USA). MSC from passages 4 to 5 were used for transplantation experiments after lentiviral transduction with a human vector expressing the enhanced green fluorescent protein (eGFP) gene which enabled *ex vivo* cell tracing via immunohistochemistry. The cells transduced with lentivirus for eGFP, transmitted the eGFP signal in the FITC channel of the FACSCanto II (BD Biosciences, San Diego, CA, USA). All sampling procedures were performed in accordance with the Helsinki Declaration and were approved by the ethics committee of Leiden University Medical Center (LUMC). All patients provided written informed consent.

*In Vitro Differentiation*

For osteogenic differentiation uMSC and iMSC were grown to 80% confluency in 24-well culture plates and were stimulated for 21 days in osteogenic differentiation medium consisting of α-MEM (Invitrogen Corp., Paisley, UK) with L-glutamin (200nM, Invitrogen Corp., Paisley, UK), P/S and 10% FCS supplemented with 10^7^ M dexamethason, 50 μg/ml Vitamin C (both from Sigma-Aldrich Chemie BV, Zwijndrecht, The Netherlands), and 5 mM β-glycerophosphate (Sigma-Aldrich Chemie BV, Zwijndrecht, The Netherlands) and stained for alkaline phosphatase activity with Fast Blue (Sigma-Aldrich Chemie BV, Zwijndrecht, The Netherlands) and for calcium deposition with Alizarine Red (MP Biomedicals LLC, Illkirch Cedex, France.) For adipogenic differentiation, MSC were stimulated for 21 days in adipogenic differentiation medium consisting of α-MEM with L-glutamin (200nM, Invitrogen Corp., Paisley, UK), P/S and 10% FCS supplemented with 10^7^ M dexamethason, insulin (10 μg/ml), indomethacin (5μM) and 3-isobutyl- 1-methylxanthine (5μM) (all from Sigma-Aldrich Chemie BV, Zwijndrecht, The Netherlands). Formation of lipid droplets was visualized with Oil-red O staining (Sigma-Aldrich Chemie BV, Zwijndrecht, The Netherlands).

*Suppression of PBMC proliferation by MSC*

Cultured human MSC were plated in graded doses in 96-well flat-bottom plates (Corning, Life Sciences) and allowed to adhere overnight. Human peripheral blood mononuclear cells (PBMC) isolated from buffy coats (1.0 x 10^5^/well) were added to the MSC and stimulated with human T-activator CD3/CD28 dynabeads (Invitrogen Corp., Paisley, UK) in a bead:cell ratio 1:5. After 5 days of co-culture, cells were pulsed with [^3^H]-thymidine (0.5 µCi/well) and incubated for 16 h at 37°C. The cultures were harvested on a glass fiber filter and thymidine incorporation was measured with a liquid scintillation counter (Wallac, Turku, Finland). Data were expressed as mean corrected counts per minute (CCPM) of triplicate co-cultures were stimulated with anti-CD28/ anti-CD3-coated Dynabeads (one bead/5 cells, Invitrogen) and were seeded in Iscove’s modified Dulbecco’s media (Invitrogen) supplemented with 5% human serum (Sanquin, Leiden, The Netherlands), and 5% FBS.

**Myocardial Infarction (MI) induction and Cell injection**

Mice received 100µL NaCl, containing 2 µg buprenorphine, subcutaneously before surgery and again 12 hours after surgery. Animals were anesthetized with 5% isoflurane for induction and kept anesthetized with 1.5-2% isoflurane in oxygen for the remainder of the surgical procedure. Mice were placed supine on a heating pad (34°C), intubated and ventilated using a rodent ventilator (model 845, Harvard Apparatus, Holliston, MA, USA) with 160 breaths per min and a stroke volume of 220 μL. A left thoracotomy was performed, followed by opening of the pericardial sac. The LAD coronary artery was visualized and ligated 1 mm caudally from the tip of the left auricle using a 7-0-prolene suture (Johnson and Johnson, New Brunswick, NJ, USA).
Ischemia was confirmed by myocardial blanching. One minute after LAD ligation animals received an introperitoneal injection of lidocaine (6mg/kg) to prevent cardiac arrhythmias [1]. Five minutes after LAD ligation animals received either 2×10^5^ uMSCs in 15 μL phosphate-buffered saline (PBS) (uMSC group), or 2×10^5^ iMSCs stimulated with IFN-γ in 15 μL PBS (iMSC group), or 15 μL PBS containing no cells (PBS group). Intramyocardial injections were performed at 3 sites in the infarcted area (5 μL per site). The chest was then closed in layers and animals were allowed to recover.
Sham-operated animals were operated in parallel, but without LAD ligation and intramyocardial injection, and were used to determine baseline characteristics (Sham group).
All surgical procedures and injections were performed by an investigator blinded to treatment.

**Cardiac Magnetic Resonance Imaging (MRI)**

Cardiac parameters were assessed 2 and 14 days post-MI using a 7-Tesla MRI (Bruker Biospin, Ettlingen, Germany) equipped with a combined gradient and shim coil, which is inserted into the magnet bore. Mice were pre-anesthetized as described above and kept anesthetized with 1.5-2% isoflurane, and placed supine in an animal holder. A respiration detection cushion was placed underneath the thorax and connected to a gating module to monitor respiratory rate (SA Instruments, Inc., Stony Brook, NY, USA). Image reconstruction was performed using Bruker ParaVision 5.1 software.

*Left ventricular function*

Cardiac function was assessed at day 2 and 14. A high-resolution 2D cine sequence was used to acquire a set of 9 contiguous 1 mm slices in short-axis orientation covering the entire heart. Imaging parameters were: echo time of 1.49 ms, repetition time of 5.16 ms, field of view (26 mm)^2^ and a matrix size of 144x192.

*Image analysis*

All MR image data were analysed with the MASS for Mice software package (Leiden, the Netherlands). The endocardial and epicardial borders were delineated manually by an investigator blinded to treatment (uMSC group n=12, iMSC group n=7, PBS group n=7, Sham group n=10). Subsequently, the end-diastolic volume EDV, end-systolic volume (ESV) and ejection fraction (EF) were computed.

**Pressure-Volume (PV) measurements**

Fifteen days after MI, mice were anesthetized again as described above and kept anesthetized with 1-1.5% isoflurane for the remainder of the surgical procedure. A 1.2F pressure-conductance catheter (standard; Scisense Inc, London, Canada) was introduced via the right carotid artery and positioned in the left ventricle (LV). The conductance catheter was connected to a PV control unit FV 896B (Scisense Inc, London, Canada) for online display and recording of LV pressure and volume signals. Parallel conductance was assessed by the hypertonic saline method using intravenous bolus injections of ~5 µL [2]. The abdomen was opened to enable temporary preload reductions by directly compressing the inferior caval vein. All data were acquired using Powerlab 8/30 Model ML870 (ADInstruments, Spechbach, Germany) and LabChart 7 software (ADInstruments, Spechbach, Germany). Data were analyzed off-line by a blinded investigator (uMSC group n=7, iMSC group n=7, PBS group n=5, Sham group n=7).
LV pressure-volume signals were acquired in steady-state to quantify general hemodynamic conditions and generate pressure-volume loops.

**Histology**

At day 15 post-MI, mice were weighed, sacrificed after PV-loop measurements under 5% isoflurane and their hearts and lungs were removed. Lungs were weighed immediately after excision, freeze-dried for 24 hours and then weighed again. The wet weight/dry weight ratio was used as a measure of pulmonary congestion.

Per group 5 hearts were fixed by immersion in buffered 4% paraformaldehyde and embedded in paraffin. Serial transverse sections of 5 μm were cut along the entire long axis of the LV for (immuno)histological analyses. Sections were deparaffinated and dehydrated in xylene and alcohol. Antigen retrieval was accomplished by heating in a microwave oven (98°C) in 0.01 M citric buffer of pH 6.0 for 12 minutes for all sections. Sections were incubated overnight at room temperature with primary antibodies and for 60 minutes with secondary antibodies.

*Engraftment rate*

MSC engraftment was detected by immunostaining with a rabbit anti-GFP antibody (A11122, Invitrogen, Paisley, UK), followed by a biotinylated goat anti-rabbit IgG (E0432, Dako, Glostrup, Denmark) and a Qdot 655 streptavidin-conjugated (Q10121MP, Invitrogen, Paisley, UK) antibody. Nuclei were visualized by Hoechst 33342 (Invitrogen, Paisley, UK).
The number of engrafted MSCs was assessed by counting the GFP-positive cells at a 20x magnification in every 10^th^ serial section along the long axis of the heart. The number of counted cells was multiplied by 10 to obtain an estimate of the total number of engrafted cells in the heart. Subsequently this number was divided by the total number of transplanted MSCs (2×10^5^) and the result was multiplied by 100%.

**Real-time PCR**

Mice were sacrificed 15 days post-MI to quantify the number of transplanted MSCs by real-time PCR [3]. Per group 5 hearts were harvested, frozen in liquid nitrogen and stored at -80° C. Hearts were minced with fine scissors and suspended in lysis buffer (100 mM NaCl, 10 mM Tris-Cl pH 8, 25 mM EDTA pH 8, 0.5% SDS, 0.1 mg/ml proteinase K) and incubated at 55° C overnight, followed by incubation in 0.025 mg/ml ribonuclease at 37° C for 1 hour. DNA concentrations were measured using NanoDrop 1000 (NanoDrop products, Wilmington, DE, USA).
PCR reactions were performed in a volume of 10 µL, containing 5 µL Universal PCR Master Mix (Applied Biosystems, Carlsbad, CA, USA), 900 nM forward and reverse primers, 250 nM TaqMan probe and 50 ng of target template. Reactions were incubated at 50° C for 2 min and 95° C for 10 min, and then amplified for 40 cycles. Each cycle comprised of an incubation step at 95° C for 15 s followed by 60 ° C for 1 min.

Standard curves were generated by serially diluting human genomic DNA (Roche, Basel, Switzerland) in murine genomic DNA. The sequence of the PCR primers and probe used for detection of human Alu repetitive sequences were as follows: forward PCR primers, 5’-CATGGTGAAACCCCGTCTCTA-3’; reverse PCR primer, 5’-GCCTCAGCCTCCCGAGTAG-3’; TaqMan probe, 5’-FAM-ATTAGCCGGGCGTGGTGGCG-TAMRA-3’.

**Flow cytometry**

From each treatment group, mice not subjected to functional and histological analysis were used for flow cytometric analysis of cardiac inflammatory cell invasion. Mice were sacrificed on days 1, 3, and 7 after myocardial infarction (*n* = 3 mice per time point). Non-operated animals were used as controls to determine base line characteristics. Infarct tissue and healthy hearts were harvested, minced with fine scissors, and placed into a solution of 2% collagenase I^A^ (Sigma-Aldrich, Chemie BV, Zwijndrecht, The Netherlands) in PBS and shaken at 37 ° C for 1 h. The cell suspension was then triturated through a nylon mesh and centrifuged in PBS at 300 g for 10 min at 4°C. Red blood cells in the cell pellet were lysed with lysis buffer (AZL, Leiden, The Netherlands), and the cells were washed in PBS and subsequently resuspended medium containing IMDM; Lonza, Verviers, Belgium) supplemented with 2.5% fetal calf serum (FCS; Greiner Bio-one) and Penicillin/Streptomycin (P/S; Invitrogen Corp., Paisley, UK). Total cardiac cell numbers were determined with a Sysmex cell counter (Sysmex America, Inc. Mundelein, Illinois, USA). The resulting single-cell suspensions were stained for flow cytometry with primary antibodies for 30 minutes at 4°C in the dark and the cells were washed with PBS/1% human Albuman (Sanquin, Leiden, The Netherlands) before analysis using a FACSCanto II (BD Biosciences, San Diego, CA, US). The following antibodies were used: anti–CD90-APC, 53–2.1,–B220-APC, RA3-6B2, –CD49b-APC, DX5, –NK1.1-APC, PK136, –Ly-6G-APC, 1A8, CD11b-eFluor 450, M1/70,–CD11c-FITC, HL3, – I-A^b^ -FITC, AF6-120.1, –Ly-6C-PE, AL-21, –CD11c-PE, HL3 (All above antibodies are from BD Biosciences), –F4/80-FITC, C1:A3-1 (ABD Serotec, Kidlington, UK). Monocytes were identified as CD11b high (CD90/B220/CD49b/NK1.1/Ly-6G) low (F4/80/I-A^b^ /CD11c) low Ly-6C high/low as previously described [4,5]. Macrophages were identified as CD11b high, F4/80 high. Dendritic cells were identified as CD11b, I-A^b^ and CD11c high. Neutrophils were identified as CD11b, Ly-6G high. Monocyte and macrophage/dendritic cell numbers were calculated as the total cells multiplied by the percentage of cells within the monocyte/macrophage gate. The analysis of the acquired data was done with FlowJo software version 7.6.1 (Tree Star Inc. Ashland, OR, USA).

**Statistical Analysis**

Numerical values were expressed as means ± standard deviation (SD). Comparison of MRI parameters and inflammatory cells between the uMSC, iMSC, PBS and Sham groups was performed using two-way repeated-measures analysis of variance (ANOVA), with Bonferroni correction. Comparison of the remaining parameters was performed using one-way ANOVA, with Bonferroni correction.

Reference List

1. Tarnavski O, McMullen JR, Schinke M, Nie Q, Kong S, Izumo S (2004) Mouse cardiac surgery: comprehensive techniques for the generation of mouse models of human diseases and their application for genomic studies. Physiol Genomics 16: 349-360. 10.1152/physiolgenomics.00041.2003 [doi];00041.2003 [pii].

2. Steendijk P, Baan J (2000) Comparison of intravenous and pulmonary artery injections of hypertonic saline for the assessment of conductance catheter parallel conductance. Cardiovasc Res 46: 82-89. S0008-6363(00)00012-2 [pii].

3. McBride C, Gaupp D, Phinney DG (2003) Quantifying levels of transplanted murine and human mesenchymal stem cells in vivo by real-time PCR. Cytotherapy 5: 7-18. 10.1080/14653240310000038 [doi];MXPHDNV0VX33RX55 [pii].

4. Nahrendorf M, Swirski FK, Aikawa E, Stangenberg L, Wurdinger T, Figueiredo JL, Libby P, Weissleder R, Pittet MJ (2007) The healing myocardium sequentially mobilizes two monocyte subsets with divergent and complementary functions. J Exp Med 204: 3037-3047. jem.20070885 [pii];10.1084/jem.20070885 [doi].

5. Swirski FK, Nahrendorf M, Etzrodt M, Wildgruber M, Cortez-Retamozo V, Panizzi P, Figueiredo JL, Kohler RH, Chudnovskiy A, Waterman P, Aikawa E, Mempel TR, Libby P, Weissleder R, Pittet MJ (2009) Identification of splenic reservoir monocytes and their deployment to inflammatory sites. Science 325: 612-616. 325/5940/612 [pii];10.1126/science.1175202 [doi].
